# The Influence of Antenna Height on the Measurement of Collective Variables Using an Ultra-Wide Band Based Local Positioning System in Team Sports

**DOI:** 10.3390/s21072424

**Published:** 2021-04-01

**Authors:** José Pino-Ortega, Asier Los Arcos, Petrus Gantois, Filipe Manuel Clemente, Fabio Yuzo Nakamura, Markel Rico-González

**Affiliations:** 1 Faculty of Sports Sciences, University of Murcia, San Javier, 30100 Murcia, Spain; josepinoortega@um.es; 2 BIOVETMED & SPORTSCI Research Group, Department of Physical activity and Sport, Faculty of Sport Sciences, University of Murcia, San Javier, 30100 Murcia, Spain; 4 Associate Graduate Programme in Physical Education, Department of Physical Education, Federal University of Paraíba, João Pessoa, Paraíba 58051-900, Brazil; pgm.gantois@gmail.com (P.G.); fabioy_nakamura@yahoo.com.br (F.Y.N.); 3 Society, Sports and Physical Exercise Research Group (GIKAFIT), Department of Physical Education and Sport, Faculty of Education and Sport, University of Basque Country (UPV-EHU), 01007 Vitoria-Gasteiz, Spain; 5 Escola Superior Desporto e Lazer, Instituto Politécnico de Viana do Castelo, Rua Escola Industrial e Comercial de Nun’Álvares, 4900-347 Viana do Castelo, Portugal; filipe.clemente5@gmail.com; 6 Instituto de Telecomunicações, Delegação da Covilhã, 1049-001 Lisboa, Portugal; 7 Research Centre in Sports Sciences, Health Sciences and Human Development, CIDESD, University Institute of Maia, ISMAI, 4475-690 Maia, Portugal; 8 Department of Physical Education and Sport, University of Basque Country (UPV-EHU), Lasarte 71, 01007 Vitoria-Gasteiz, Spain

**Keywords:** EPTS, athlete tracking, position, team behavior, sport

## Abstract

Ultra-wide band (UWB) based local positioning systems (LPS) are based on devices and a portable antenna set. The optimal installation height of the antennae is crucial to ensure data accuracy. Collective variables are metrics that consider at least two pairs of coordinates, which may lead to lower precision than an individual one. Therefore, the aim of this study was to compare the influence of antenna height with collective metrics using a UWB (i.e., IMU; WIMU PRO™, RealTrack Systems, Almeria, Spain) based LPS. Data acquisition was carried out in a basketball court measuring 28 × 15 m. Five devices were used; one of which was carried by a healthy and well-trained athlete (age: 38 years, mass: 76.34 kg, height 1.70 m), while each of the remaining four was positioned on a tripod in one of the four corners of the court. Four kinds of variables were extracted: (1) static distances, (2) dynamic distances, (3) static areas and (4) dynamic areas in all antenna installation modes of 0.15, 1.30 and 2.00 m. The results showed that the antenna of 1.30 m provided better accuracy for all measures (% difference range from −0.94 to 1.17%) followed by the antenna of 2.00 m (% difference range from −2.50 to 2.15%), with the antenna of 0.15 m providing the worst accuracy level (% difference range from −1.05 to 3.28%). Overall, the measurements of distance metrics showed greater accuracy than area metrics (distance % difference range from −0.85 to 2.81% and area % difference range from −2.50 to 3.28). In conclusion, the height of the antennae in basketball courts should be similar to the height at which the devices are attached to a player’s upper back. However, as the precision is sensitive to the magnitude of the measure, further studies should assess the effects of the relative height of antennae in team sports with greater playing spaces.

## 1. Introduction

The calculation of positional data, which is based on the location of a player and represented by geographic or Cartesian coordinates [1], has enabled the measurement of a wide range of tactical behavior variables in team sports [2,3,4,5]. These variables can be clustered into individual (i.e., the measurement of the behavior of a player in isolation) [6] and collective (e.g., variables that measure the relationship between several players such as the mean distance between players and the space occupied by the team) metrics [3,4]. In addition, collective tactical behavior can encompass variables according to geometric primitives: (a) geometrical center (GC) or point [2,5], distance [3] and area [4]. These variables can be used to carry out a linear analysis to assess the mean position of a team, the distance between the positioning of two points (i.e., player, GC, goal, lines) and a team’s space occupation or distribution in the field. In addition, non-linear analysis techniques (i.e., relative phase and entropy) enable the examination of the coordination between the motion of two oscillators (i.e., players, GCs) and the predictability of the movements [2,3,5,7]. Based on these data, technical staff can evaluate tactical behavior during the training process and the competition in team sports [7,8]. As the movement of a single player in a team sport can functionally influence the spatial-temporal characteristics of coordinated movements of teammates and opponents [9] and considering that the non-relationship between individual variables and success has been highlighted [10,11], collective metrics have gained relevance in team sports [2,3,5].

The analysis of positional data and, subsequently, of collective metrics, is fully available thanks to electronic performance and tracking systems (EPTS). Among the different tracking systems, local positioning systems (LPS) have been highlighted as the most accurate system to measure collective tactical behavior in team sports [12,13,14]. Specifically, between different technologies in which LPS are based (i.e., ultrasound, ultra-wide band (UWB)), UWB seems to be the most promising technology due to the bandwidth and possibility of operating in multipath environments [15,16,17] where even with 28 devices turned on it has shown a high accuracy [18]. LPS consist of a reference system composed of a set of antennae and a portable device attached to tight-fitting garments on the backs of the athletes [17]. Considering the known position of the reference system, LPS calculate each player’s position using an algorithm, through which the distances between the antennae and player are extracted [15,16,19]. The accuracy of this computation, which is defined by the Joint Committee for Guides in Metrology as the closeness of agreement between an estimated position and a true quantity value of a measure [15], is a crucial factor to be considered when using this technology. Previous work has highlighted different configurations (e.g., the shape of the antennae installation, measurement methods) that can affect the quality of the measure using LPS, one of which is the height of the antennae [17].

### Related Works: The Novelty of the Proposal

In sports, the relative height of antennae has been set differently across studies; i.e., between 1.6 m and 1.85 m [18,20], at 3 m [14,21] and at 4.8 m [22]. There is no consensus about the antenna height even between the same UWB based LPS [18,20]. In this respect, it has been theoretically proposed that the higher the antennae are, the higher the measurement errors associated with the positional data will be [17]. However, this fact has not been sufficiently investigated. To the authors’ knowledge, only one study has assessed the influence of relative antenna height in a sport setting [23]. Martinelli et al. [23] assessed the effects of the relative height of a UWB technology based system antenna set (i.e., four antennae). These authors set the antennae at heights of 1.00 m, 1.60 m and 2.00 m from the floor and looked for the most suitable antenna height during athlete motion. The results suggested that the height of the antennae should be similar to the height at which the device is located on a player’s upper back (i.e., around 1.6 m height) [23]. As this study considered individually analyzed athlete movements, did not consider collective metrics and highlighted that a slight error in the computation of a player position may cause a substantial deviation from the gold standard in a collective metric, it seems to be of interest to assess the influence of the height of antennae on the measurement of collective variables [12]. Therefore, the current study aimed to compare the influence of antenna height with the measurement of collective variables (i.e., distances and areas).

## 2. Method

### 2.1. Experimental Approach to the Problem

In a recently published survey [17], the methodological use of EPTS was detailed. Specifically, the use of LPS was summarized into twenty-three items where the height of antennae was one of them. The antennae are usually installed in different team sport facilities by a sport scientist [12,24,25] instead of by providers. Thus, the assessment of the influence of the height of the antennae for the measurement of tactical variables may help sport analyst professionals to record information with a greater quality of the data. As it has only been assessed one time previously [23] a further analysis to corroborate the findings is necessary. In this way, collective variables are more sensible because they can report an accumulative error arisen from the computation of each player positioning [12]. In addition, the greater magnitudes that suppose collective variables other than time-motion variables may enhance the possible reported bias with different installation heights. Looking at this research question, four kind of variables (i.e., static and dynamic distances and static and dynamic areas) were recorded using three different antennae heights: (i) a protocol with the UWB’s antenna height at 0.15 m, (ii) a protocol with the UWB’s antenna height at 1.30 m and (iii) a protocol with the UWB’s antenna height at 2.00 m.

### 2.2. Participants

A healthy and well-trained athlete (age: 38 years, mass: 76.34 kg, height 1.70 m) volunteered to participate in the current investigation. Subject height was measured using a stadiometer (SECA, Hamburg, Germany) and body mass was obtained using a scale (TANITA BC-601, Tokyo, Japan). The participant did not present any physical limitations or musculoskeletal injuries that could affect the testing. The study was conducted according to the Declaration of Helsinki and was approved by the Bioethics Commission of the University of Murcia (ID: 2061/2018). Participants were informed of the risks and provided written informed consent.

### 2.3. Technology

The study methodology on the use of EPTS was written following the protocol suggested by Rico-González, Los Arcos et al. [17] in order to guarantee a precise description of the use of technology. In total, the score was 21 points out of 23 (91%). The items general criteria 8 and general criteria 11 could not be explained because the authors did not have information about these items.

#### 2.3.1. Tracking System

Positional data in a court were recorded with a time-motion tracking system using five inertial measurement units (IMU; WIMU PRO™, RealTrack Systems, Almeria, Spain). Each device had an internal microprocessor, a 2 GB flash memory and a high-speed USB interface to record, store and upload data. The devices were powered by an internal battery with 4 h of life, had a total weight of 70 g and dimensions of 81 × 45 × 16 mm. Each device contained, among other sensors, a 33 Hz ultra-wide band (UWB) sensor. This model was certified by an International Match Standard and Quality certificate provided by the Fédération Internationale de Football Association (FIFA), which ensured that the device was safe and valid. In addition, the validity and reliability of these UWB based LPS has recently been positively evaluated [26].

#### 2.3.2. Data Recording

WIMIPRO™ inertial devices (IMU; WIMU PRO^TM^, RealTrack Systems, Almeria, Spain) computed the positioning data in the receivers. All antennae had a common clock and the receiving node performed positioning data through the time difference of arrival (TDOA) of the incoming signal and directly calculated its distance from the transmitter; thus, multiplying the estimated TDOA by the speed of light made it possible to draw a circle with the reference node at its center and a radius equal to the estimated range. By collecting at least three measurements (triangulation) and intersecting the defined circles, it was possible to determine the position of the receivers with high accuracy [15,17]. The UWB system was adjusted to the reference field before the start of the investigation.

#### 2.3.3. Data Processing

Recorded data were transformed into raw position data (x and y coordinates) using software (S PRO, RealTrack Systems, Almeria, Spain). The data were downloaded after the session because the data monitored in real-time were significantly inaccurate relative to the post-session data [27]. The reference system to compare the results was projected in the software using a geographic information system (GIS) mapping application. A GIS allows the representation of geometrical shapes such as polygons or circles with millimeter accuracy. In this way, the routes selected with their real measurements (measured by a tape measure) were introduced to the previously created template. Subsequently, the x and y coordinate data of the UWB system were introduced and compared. The distance error of each axis was reported. Of all of the data entered, only those that corresponded to the execution of the routes were selected.

### 2.4. Procedure

Data acquisition was carried out in a basketball court measuring 28 × 15 m. The conditions were maintained with temperatures at 20 °C, low humidity gradients (55%) and slow air circulation (<5 km/h) to allow for easier positioning [15]. As the UWB could be subject to interference caused by metallic materials [15], the protocol was carried out in a location distant from this kind of material.

**Figure 1:**
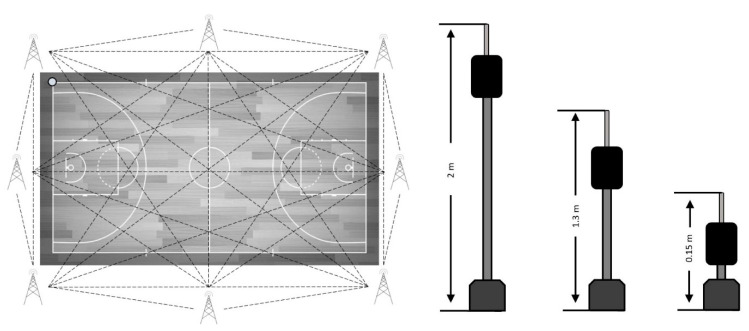
The ultra-wide band (UWB) reference system setting and height of antennae.

The reference system was composed of eight antennae placed around the field. The antennae with the UWB technology were fixed 2 m from the perimeter line in the corners (*n* = 4) and 3.5 m from the middle line of the field (*n* = 2) and from behind the goals (*n* = 2) (Figure 1), forming an octagon for better signal emission and reception [26]. The antennae installation was performed on three occasions at three different heights from the floor: 0.15 m, 1.30 m and 2.00 m (Figure 1). Once installed, the units were switched on one by one with the master antenna turned on last. From that moment, it was necessary to respect a 5 min protocol in which the computing nodes in the antennae calculated their positioning and the distance between them. This protocol was performed after setting the height configuration of each antenna.

**Figure 2:**
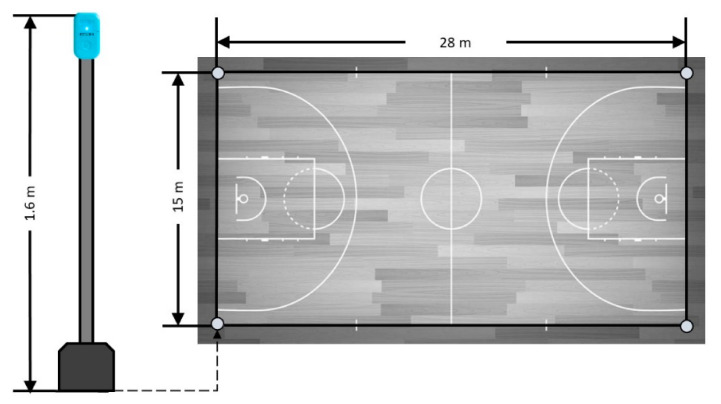
The UWB reference system setting and height of antennae.

Five devices were considered. One was attached to the player in a pocket of an appropriately tight-fitting garment placed between the scapulae at the second to the fourth thoracic vertebra (T2–T4) level (1.6 m) to avoid unwanted movements and before on-court exercises, following previous study protocols [26]. Each of the remaining devices (*n* = 4) was held by a tripod in each corner of the court at the same height as that on the player’s upper back (Figure 2). Even though five devices were simultaneously turned on, a previous study did not report any problems in UWB based tracking system accuracy with 28 devices turned on [28].

From these devices, both static and dynamic collective metrics (measures from more than one device) were extracted (see Table 1).

### 2.5. Statistical Analysis

Descriptive statistics were calculated for all variables and reported as a mean and standard deviation. The accuracy of the data measurements of distance and area was calculated as the mean difference and percentage difference (% difference) recorded by three different antennae heights (0.15, 1.30 and 2.00 m) with respect to their real distance and area measured as reference criteria. Moreover, the magnitude of the accuracy between antenna heights was adopted considering a % difference threshold using the real measures as reference criteria based on previous findings [18] as follows: better accuracy (% difference < 1; green); good accuracy (1 > % difference ≤ 2; yellow); worse accuracy (% difference > 2; red). The analyses were performed in the statistical package for the social sciences (SPSS, IBM^®^ corporation, Armonk, NY, USA), version 20.0.

## 3. Results

### 3.1. Static Distances

**Figure 3:**
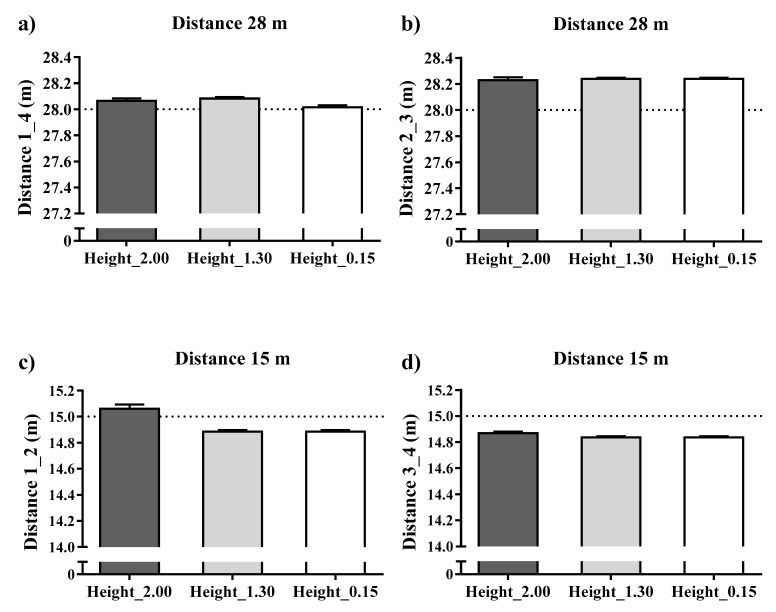
Accuracy in mean differences of a static UWB system for the total distances recorded by three antenna heights in meters. Note: devices installed in all corners held by a tripod were allocated numbers from 1 to 4. From these, four distances were computed: (**a**): from device 1 to device 4 (distance 1–4); (**b**): from device 2 to device 3 (distance 2–3); (**c**): from device 1 to device 2 (distance 1–2) and (**d**): from device 3 to device 4 (distance 3–4). Dashed line represents the real measures as reference criteria.

Figure 3 shows the accuracy of the static UWB system for total distance using the real distance measured as the reference criteria between the three antenna heights (0.15, 1.30 and 2.00 m). Overall, statistically, the UWB system showed a low to moderate mean and percentage difference values for the total distance recorded by the three antenna heights (mean bias and % difference ranged from −0.017 to 0.17 m and −0.064 to 1.1%, respectively). Specifically, the static total distance recorded by the antennae heights of 1.30 and 0.15 m showed the worst mean and percentage differences (bias = 0.16 ± 0.01 m and % difference = 1.1 ± 0.06%).

### 3.2. Static Areas

**Figure 4:**
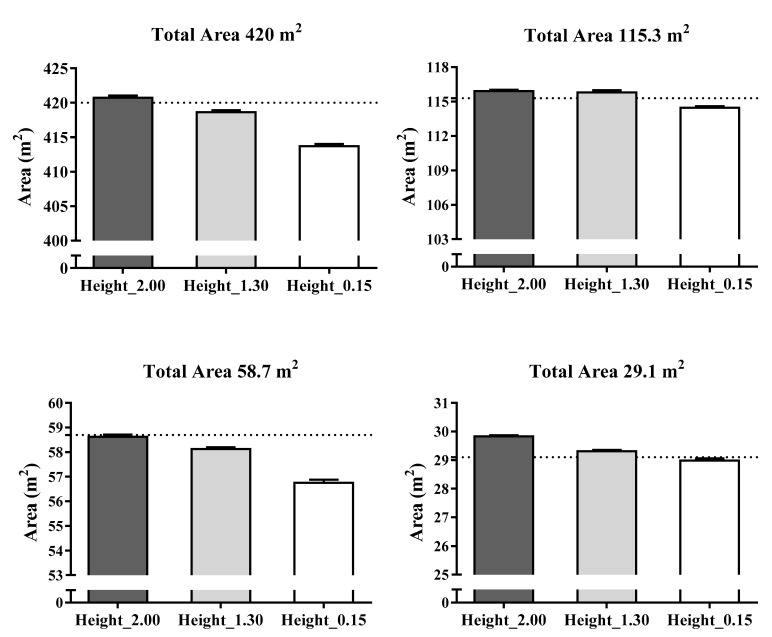
Accuracy in mean differences of a static UWB system for the total area recorded by three antenna heights in meters. Dashed line represents the real measures as reference criteria.

Figure 4 shows the accuracy of the static UWB system for determining different total areas recorded by three antenna heights (2.00, 1.30 and 0.15 m). Overall, the mean and percentage differences in data measurements of the total area using the real areas as reference criteria ranged from −0.08 to −6.33 m and −0.08 to 3.28%, respectively. Specifically, the average bias for the total area recorded by the 0.15 m height antenna presented the worst values for the 420 m^2^ (mean bias = −6.33 ± 0.35 m^2^ and % difference = −1.51 ± 0.08) and 58.7 m^2^ areas (bias = −2.00 ± 0.12 m^2^ and % difference = 3.28 ± 0.21%). In addition, the total area recorded by the antenna height of 2.00 m showed mean and % differences of 0.72 ± 0.04 m^2^ and 2.54 ± 0.15% for the 29.1 m^2^ area, respectively.

### 3.3. Dynamic Distances

**Figure 5:**
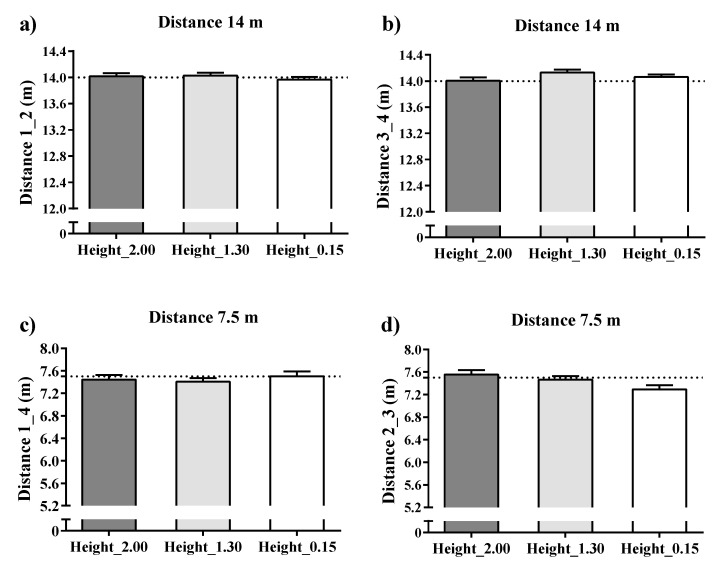
Accuracy in mean differences of a dynamic UWB system for the dynamic distances recorded by three antenna heights in meters. Note: devices installed in all corners held by a tripod were allocated numbers from 1 to 4. From these, four distances were computed: (**a**): from device 1 to device 2 (distance 1–2); (**b**): from device 3 to device 4 (distance 3–4); (**c**): from device 1 to device 4 (distance 1–4) and (**d**): from device 2 to device 3 (distance 2–3). Dashed line represents the real measures as reference criteria.

Figure 5 presents the accuracy of the dynamic total distance (goal to goal and side to side directions) in mean and percentage differences of the real total distance measured and recorded by the UWB system. Overall, the dynamic UWB system showed low mean difference values for the total distance recorded by the three antenna heights (mean bias and % difference ranged from −0.003 to 0.21 m and 0.02 to −1.14%, respectively). The total dynamic distance recorded by the 0.15 m antenna height showed the worst values for distance 2–3 (7.5 m) (bias = 0.21 ± 0.08 and % difference = 2.81 ± 1.04%).

### 3.4. Dynamic Areas

**Figure 6:**
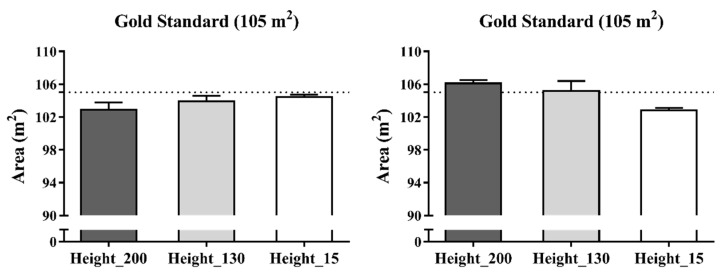
Accuracy in mean differences of a dynamic UWB system for the total area recorded by three antenna heights in meters. Note: Dashed line represents the real measures as reference criteria.

Figure 6 presents the accuracy of the dynamic UWB system for determining goal to goal and side to side total area according to the three antenna heights (2.00, 1.30 and 0.15 m). Overall, the mean and percentage differences in data measurements of the total area using the real areas as reference criteria ranged from −0.21 to 2.26 m^2^ and −0.20 to 2.15%, respectively. Specifically, the worst mean and percentage differences were for the antenna height of 2.00 m in the goal to goal total area (mean bias = −2.26 ± 0.84 and % difference −2.15 ± 0.80%) and for the antenna height of 0.15 m in the side to side total area (bias = −2.16 ± 0.26 and % difference = 2.06 ± 0.24).

### 3.5. Overall Results

**Figure 7:**
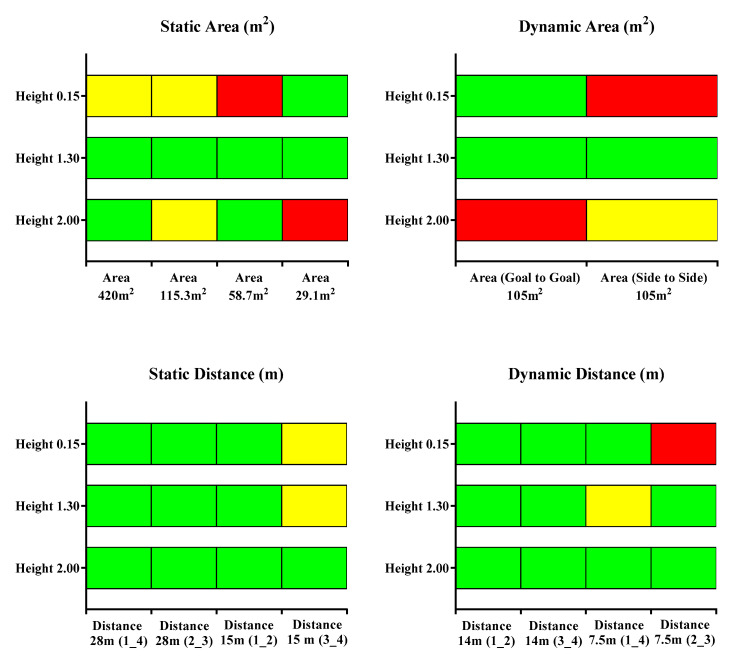
Overall accuracy in percentage terciles (green < 1%, yellow = 1–2%, red > 2%) of a static and a dynamic UWB system to determine the distance and total area in the three antenna heights in meters.

Figure 7 presents the overall accuracy for the position data as the % difference of the distance and total area using the real measurements as reference criteria. Each color represents the % difference threshold (green < 1%, yellow = 1–2%, red > 2%). Overall, data recorded by the 1.30 height antennae showed lower % differences for all measures followed by the 2.00 m height antennae, with the 0.15 m height antennae displaying the worst accuracy levels. Moreover, better accuracy values were found for the total distance in comparison with the total area.

## 4. Discussion

This study aimed to compare the influence of antennae height with the measurement of the collective metrics (i.e., distance and area variables) using UWB based LPS. The main finding was that the accuracy of the data to measure distances and areas representative of collective variables was greater when the height of the antennae were 1.30 m in comparison with 2.00 and 0.15 m. This suggested that the height of the antennae should be similar to the height at which the device is attached to a player’s upper back during the measurement of tactical behavior in sport.

Recently, the number of manufacturers that offer LPS has grown exponentially [19,29] although each of them offers technologies that differ in a wide range of characteristics [19]. For practical purposes, EPTS should be valid tracking methods that are understood as their capacity to extract a measure with a high proximity from the true value that they are intended to measure [19]. In sport, it has been suggested that a difference lower than 15–20 cm between the true and measured values makes a technology valid under dynamic conditions [30]. However, the characteristics of the test designed (i.e., intensities, change of direction) to assess the validity of a technology can differ widely, influencing the outcomes [19,31]. Therefore, a standardized validity test is of interest. In this sense, Link, Linke and Lames [31] proposed a standard using three validity tests (i.e., three levels): (i) a static condition, (ii) a velocity test and (iii) continuous situations. Similarly, the Fédération Internationale de Football Association (FIFA) has tried to propose a standardization option, classifying tests into two main categories: (i) a dynamic test and (ii) a continuous situation test (i.e., 2 vs. 2, 3 vs. 3, 5 vs. 5 and full pitch coverage of small-sided games). It should be highlighted that although static assessment could be considered to be a preliminary evaluation, dynamic tests are required as the main basis for classifying the technology as valid. In fact, static tests were mainly used some years ago when EPTS began to be applied in sport settings [32,33]. For example, Frencken, Lemmink and Delleman [33] assessed the accuracy of 25 static transponders (22 placed on the pitch and three on a tight-fitting garment worn by players) and found mean errors of 1 ± 0 cm and 2 ± 1 cm, respectively. These analyses, together with other situations, were considered useful in making the technology commercially available after which these studies were cited in other empirical studies to track data during small-sided games and matches [24,25]. However, as tracking systems are designed to record data during training and matches, the assessment of static coordinates lacks a similarity to reality in sports.

The wide interest in training and competition load variables has led to the design of accuracy tests to validate player movement tracking systems in isolation, resulting in individual variables such as distance covered at different intensities [19,34,35]. It has been highlighted that an individual player’s motion can affect the spatial-temporal dynamics of the remaining players involved in a game [9] and that there is a lack of a relationship between individual variables and success [10,11] leading to collective metrics gaining relevance in team sports [2,3,5]. However, the tests designed to validate technology for collective metrics only allow a comparison (or testing the agreement) by using another validated technology or gold standard, highlighting the necessity of tests that enable the determination of the validity of tracking systems for collective variables [12,13]. The main reason to propose these tests was supported by Rico-González et al. [12] who reported that as a collective metric is a computation of a unique value from more than one player, if a system has a bias in a measure of a single player, it could tend to be greater in collective settings. Therefore, if the static coordinate computation could be of interest and time-motion variables are necessary to make a technology valid, the new trends should consider the design of validity tests to extract collective metrics.

In sport, the validity study is a widely extended topic, clustering thousands of publications that have resulted in different reviews [19,34,35,36,37]. These articles are likely the results of continuous modifications that manufacturers perform in their hardware and software. This fact resulted in a new trend of research [17] aimed at testing methodological aspects and specific characteristics of devices such as sampling rates, algorithms, the number of antennae and antenna installation as main factors that could influence the accuracy of the derived measures. Recently, the main factors that may influence data quality using local positioning systems have been highlighted, e.g., the turn off process, data transmission and downloading, validity and reliability issues, calculation algorithms, the sampling rate, environmental conditions, infrastructure conditions and installation issues [17]. A few of these issues such as the sampling rate [38] or data transmission and downloading [27] have been assessed while others remain unknown. One of the factors clustered into installation issues is the height of antennae and, in sport settings, LPS have been used with very different ranges of antenna height [14,18,21,22,28,30,39]. It has been suggested that each antenna assumes an error around it and the higher the antennae are, the greater the associated errors will be [17]. Moreover, it has been suggested that the height of the UWB anchor installation should be similar to the height of the antennae [18]. However, only one study has assessed this question empirically during an individual assessment [23]. For this reason, the present study aimed to evaluate LPS during both static and dynamic conditions for collective metrics.

### 4.1. Static Collective Metrics

In the present study, the tested UWB was shown to be valid and accurate in the measure of the distance between two devices at least during non-static drills (Figure 3). Moreover, this study evidenced that the accuracy for static distances was dependent on antenna height, highlighting that a height of 1.30 m was the most appropriate in practical sports settings against 2.00 and 0.15 m, which showed the worst mean and percentage differences (bias = 0.16 ± 0.01 m and % difference = 1.1 ± 0.06). In addition, the inclusion of a static assessment of areas showed that the bias and percentage of error were −0.08 to −6.33 m^2^ and −0.08 to 3.28%, respectively. However, while analyzing the influence of the height of the antennae, it seemed that installation height could affect the outcomes, mainly in area measures. Specifically, the average bias for the total area recorded by the antennae with a height of 0.15 m presented the worst values for the 420 m^2^ (bias = −6.33 ± 0.35 m^2^ and % difference = −1.51 ± 0.08) and 58.7 m^2^ areas (bias = −2.00 ± 0.12 m^2^ and % difference = 3.28 ± 0.21) compared with the other two heights. In addition, the total area recorded by the antennae height of 2.00 m showed mean and % differences of 0.72 ± 0.04 m^2^ and 2.54 ± 0.15 for the 29.1 m^2^ area, respectively. Unfortunately, to the authors’ knowledge, these results cannot be compared with others because no studies have been proposed in which the height of the antennae was assessed for collective metrics, motivating further research on this topic. Leser, Baca and Ogris [16] reported that the error of the estimation should be lower than the natural balance of the center of gravity of the human body, which considered 15–20 cm, the static distance and area variables calculated in this study seemed to present an overall acceptable accuracy at least when using an antenna height of 1.30 m. However, the nature of the game makes player tracking during motion mandatory, meaning these results were valid far from real situations.

### 4.2. Dynamic Collective Metrics

Previous studies have assessed the validity of EPTS for collective variables, proposed small-sided games [14] and official matches [12]. In both studies, the authors concluded that the assessed UWB (IMU; WIMU PRO™, RealTrack Systems, Almeria, Spain) was a suitable technology to assess collective metrics. However, these studies were aimed at assessing the agreement between two technologies, meaning that the study designs were not suitable for classifying the systems as valid. As a result, the current study was the first to enable a validity assessment of a technology for collective metrics during motion. Overall, the bias and % difference for distances ranged from −0.003 to 0.21 m and 0.02 to −1.14%, respectively. However, this seemed to differ depending on the height of antennae. Specifically, using a 0.15 m height provided the worst values for distance between device numbers 2–3 (7.5 m) (bias = 0.21 ± 0.08 and % difference = 2.81 ± 1.04) while, in general, heights of 1.30 m and 2.00 m similarly affected measures. Likewise, a general bias and % difference for areas ranged from −0.21 to 2.26 m^2^ and −0.20 to 2.15%, respectively. Specifically, the worse mean and percentage differences were for the antenna height of 2.00 m in the goal to goal total area (bias = −2.26 ± 0.84 and % difference −2.15 ± 0.80) and for the antenna height of 0.15 m in the side to side total area (bias = −2.16 ± 0.26 and % difference = 2.06 ± 0.24). In brief, the results displayed in this study revealed that the evaluated UWB was valid, mainly with an antennae height installation of 1.30 m.

### 4.3. Antenna Height: A Comparison with Previous Studies

To our knowledge, only one previous study assessed the effects of the height of antennae using LPS [23]. Martinelli et al. [23] looked at the effects of the relative height between the transmitting and the receiving antennae in each athlete’s dynamic position on a five-a-side football pitch (39 × 20.2 m). The results showed that a 1.6 m antenna height displayed the same RMSE (root mean square error) (<31 cm) as a 2.00 m height antenna (RMSE < 31 cm) and lower than a 1.00 m height antenna (RMSE < 34 cm). However, the authors recommended the medium height antenna (1.6 m) because other factors such as the loss of transmitted packets were greater using a higher antenna installation (2.00 m). It seems that the heights of UWB based LPS antennae not over the athlete’s height may be a suitable installation reference, showing that an excessively low height (<1.00 m) is not suitable [23]. This fact was consistent with the results highlighted in the present study where a height of 0.15 m showed a lower precision (>2%) in more cases than the other heights. In addition, these studies (i.e., Martinelli et al. [23] and the present study) corroborated the hypothesis that an excessively high antenna height may negatively influence the quality of the data due to the circumference of error created around each antenna [17]. As the infrastructure around antennae installation and weather conditions may affect the accuracy of a UWB [15,16,17], further investigations should be carried out in other environments and with different conditions.

The metrics considered in the study carried out by Martinelli et al. [23] were individuals, which meant that the accuracy was taken from a pair of coordinates (i.e., x and y Cartesian coordinates). Collective variables, which are the result of the position of all players involved in a certain collective metric, may report greater differences between the true value and the measure in comparison with individual variables [12]. This fact could be due to a slight deviation from the gold standard in a pair of coordinates (the positioning of a player) being added to the error in the measure of another pair of coordinates and, subsequently, a collective metric could show a lower accuracy than an individual one. Interestingly, among all of the situations considered in the current study, distance based metrics showed a higher precision in most situations in comparison with the area related measures (see Figure 7). This fact may suggest that a greater magnitude of a certain measure may affect precision when UWB antennae are installed at heights of 2.00 m and 0.15, while the magnitude seems not to have an affect using an installation height of 1.30 m.

## 5. Conclusions

Among the new EPTS validation articles, it is a trend to assess the methodology of the use of EPTS and although a few factors have already been investigated, the height of antennae for collective metrics has not yet been assessed. In this sense, the height at which the devices are placed on the athlete’s back (from 1.3 m to 1.6 m) may be a suitable reference for antenna installation in team sports with smaller playing spaces (e.g., a five-a-side football pitch, a basketball court). However, as it seems that a measure is more sensible measuring variables with greater magnitudes, further studies should assess whether greater spaces in team sports (e.g., soccer, rugby, hockey) allow greater distances between players and/or occupied areas, resulting in a requirement of different antenna heights. In this way and considering that the previous protocols have only allowed the comparison between two technologies (agreement) to measure collective metrics, future studies could use the presented protocol to assess the validity of LPS with different heights of antennae.

## Figures and Tables

**Table 1:** Variables.

Variable	Real Distance	Description	Graphic Representation
		Variables without motion (static)	
Distance	15 m	Distance between two devices located behind the same goal line (two distances).	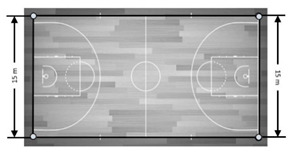
	28 m	Distance between two devices located on the same side (two distances).	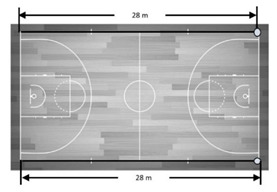
Area	420 m^2^	The whole area of the court. The gold standard (standard dimensions of a basketball court) was calculated multiplying length × width. The recorded measure was then compared with the result of this calculation (420 m^2^).	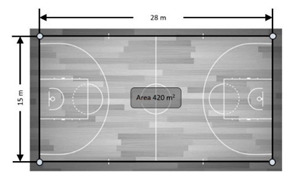
	115.29 m^2^	The area of the medium perimeter delimited in the graphic. Using a tape measure the area’s dimensions were calculated. The results of this multiplication (115.29 m^2^) were then compared with the measure.	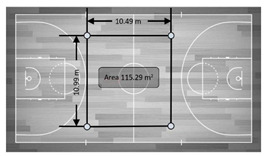
	58.74 m^2^	The area of the small perimeter delimited in the graphic. Using a tape measure the area’s dimensions were calculated. The results of this multiplication (58.74 m^2^) were then compared with the measure.	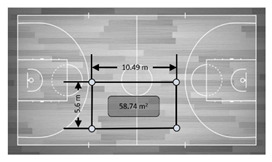
	29.10 m^2^	The area of the very small perimeter delimited in the graphic. Using a tape measure the area’s dimensions were calculated. The results of this multiplication (29.10 m^2^) were then compared with the measure.	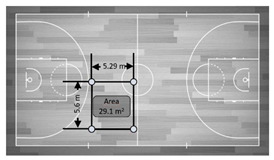
		Variables with motion (dynamic)	
Distance	7.5 m	The player moved through the dashed line (line in goal to goal direction). The distance between the athlete’s positioning and the sidelines was calculated and compared with the gold standard (gold standard = basketball court’s width (15 m)/2 sides = 7.5 m between the player and each sideline).	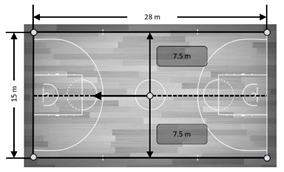
	14 m	The player was moving through the dashed line (middle line in a side to side direction). The distance between the athlete’s positioning and the background lines was calculated and compared with the gold standard (gold standard = basketball court’s length (28 m)/2 sides = 7.5 m between the player and each background line).	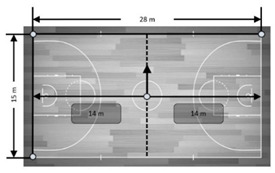
Area	105 m^2^	− Middle court (1/2 of the total court) rectangle measure: − 28 m (court’s length) × 7.5 m (basketball court’s middle width) = 210 m^2^. − Triangle measure (the measure of ¼ of the total court): − 210 m^2/^2 = 105 m^2^. − Comparison: − The continuously recorded measure from the devices was compared with a tape measure (105 m^2^).	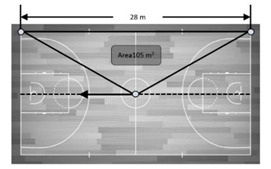
	105 m^2^	− Middle court (1/2 of the total court) rectangle measure: − 15 m (court’s width) × 14 m (basketball court’s middle length) = 210 m^2^. − Triangle measure (the measure of ¼ of the total court): − 210 m^2^/2 = 105 m^2^. − Comparison: − The continuously recorded measure from the devices was compared with a tape measure (105 m^2^).	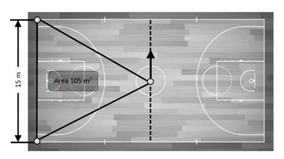

## Data Availability

Not applicable.
